# Gene-Based Testing of Interactions in Association Studies of Quantitative Traits

**DOI:** 10.1371/journal.pgen.1003321

**Published:** 2013-02-28

**Authors:** Li Ma, Andrew G. Clark, Alon Keinan

**Affiliations:** 1 Department of Biological Statistics and Computational Biology, Cornell University, Ithaca, New York, United States of America; 2 Department of Molecular Biology and Genetics, Cornell University, Ithaca, New York, United States of America; Dartmouth College, United States of America

## Abstract

Various methods have been developed for identifying gene–gene interactions in genome-wide association studies (GWAS). However, most methods focus on individual markers as the testing unit, and the large number of such tests drastically erodes statistical power. In this study, we propose novel interaction tests of quantitative traits that are gene-based and that confer advantage in both statistical power and biological interpretation. The framework of gene-based gene–gene interaction (GGG) tests combine marker-based interaction tests between all pairs of markers in two genes to produce a gene-level test for interaction between the two. The tests are based on an analytical formula we derive for the correlation between marker-based interaction tests due to linkage disequilibrium. We propose four GGG tests that extend the following *P* value combining methods: minimum *P* value, extended Simes procedure, truncated tail strength, and truncated *P* value product. Extensive simulations point to correct type I error rates of all tests and show that the two truncated tests are more powerful than the other tests in cases of markers involved in the underlying interaction not being directly genotyped and in cases of multiple underlying interactions. We applied our tests to pairs of genes that exhibit a protein–protein interaction to test for gene-level interactions underlying lipid levels using genotype data from the Atherosclerosis Risk in Communities study. We identified five novel interactions that are not evident from marker-based interaction testing and successfully replicated one of these interactions, between *SMAD3* and *NEDD9*, in an independent sample from the Multi-Ethnic Study of Atherosclerosis. We conclude that our GGG tests show improved power to identify gene-level interactions in existing, as well as emerging, association studies.

## Author Summary

Epistasis is likely to play a significant role in complex diseases or traits and is one of the many possible explanations for “missing heritability.” However, epistatic interactions have been difficult to detect in genome-wide association studies (GWAS) due to the limited power caused by the multiple-testing correction from the large number of tests conducted. Gene-based gene–gene interaction (GGG) tests might hold the key to relaxing the multiple-testing correction burden and increasing the power for identifying epistatic interactions in GWAS. Here, we developed GGG tests of quantitative traits by extending four *P* value combining methods and evaluated their type I error rates and power using extensive simulations. All four GGG tests are more powerful than a principal component-based test. We also applied our GGG tests to data from the Atherosclerosis Risk in Communities study and found five gene-level interactions associated with the levels of total cholesterol and high-density lipoprotein cholesterol (HDL-C). One interaction between *SMAD3* and *NEDD9* on HDL-C was further replicated in an independent sample from the Multi-Ethnic Study of Atherosclerosis.

## Introduction

Genome-wide association studies (GWAS) have identified over six thousand single-nucleotide polymorphisms (SNPs) associated with complex human diseases or traits [1]. Most of these SNPs have small effect sizes, and for most traits collectively explain only a small fraction of heritable genetic variance [2], [3], [4]. Epistasis has been hypothesized to play an important role in the genetic basis of complex diseases and other complex traits [5], [6], [7] and to be one of the contributors to this problem of “missing heritability” [3], [8], [9]. Even if epistasis explains only a tiny fraction of “missing heritability”, the importance of revealing the specific gene-gene interactions that underlie that fraction is also in the unique type of biological insight that gene-gene interactions can provide, i.e. from the light they can shed on the pathway level. Although many gene-gene interactions have been identified in non-human organisms [10], [11], [12], their detection and replication in human GWAS are still proving difficult (*e.g.* [13]). Challenges include the computational complexity arising from the large number of pairwise or higher-order tests when each pair or group of SNPs is considered, the extensive burden of multiple-testing correction they entail [6], [9], and the reduced statistical power of each test when applied to tag SNPs [9], [14], [15]. Several computer programs [16], [17], [18], [19], [20], [21] and statistical methods [15], [22], [23], [24], [25], [26], [27] have been developed for detecting and replicating gene-gene interactions in GWAS while addressing these challenges. In this study, we aim to improve the power of gene-gene interaction testing by moving beyond testing between a pair (or a group) of individual SNPs, which is the case in conventional marker-based testing, and instead considering all pairs of SNPs from each of a pair of genes in a single gene-based test of interaction.

Gene-based tests have been proven successful for regular GWAS tests of main (marginal) associations [28], [29], [30], and there are several potential advantages to extending this methodology to testing for gene-gene interactions. First, a gene-based approach substantially reduces the burden of multiple-testing correction, *e.g.* for 20,000 genes, there are ∼2×10^8^ possible pairwise gene-based interaction tests, while for 3 million SNPs there are over ∼5×10^12^ possible marker-based interaction tests. Second, gene-based interaction tests can increase power by aggregating signals across variants in the target regions (a gene or any other locus) when multiple causal interactions influence the phenotype of interest, as has been shown to be the case for GWAS tests of main association effects [31], [32]. Third, in cases where the interacting variants are only tagged, rather than directly observed, such tests can aggregate signals from different tag SNPs in partial linkage disequilibrium (LD) with the causal variants and with each other. Fourth, a gene-based interaction test is a natural choice when testing is focused on a reduced set of pairs based on prior biological knowledge, which is often on a gene-level, *e.g.* testing pairs of genes that exhibit protein-protein interactions (PPI) or that participate in the same pathways [15], [33], [34], [35], [36], [37]. Finally, going beyond genotype-based GWAS, gene-based interactions tests can also improve power in sequencing-based association studies, with their design being especially well-matched for whole-exome sequencing.

A gene-based interaction testing approach can also improve the power of replicating interactions that is reduced due to population heterogeneity in LD patterns leading to different tag SNP-pairs being linked to the same underlying causal interaction [15]. The power of replicating a marker-based interaction test, much like the replication power of main effects, decreases with decreasing LD between tag SNPs and the causal variants. However, for gene-gene interaction testing the observed effect size decreases by the product of LD in the two loci, therefore the reduction in power can be much greater [14], [15]. In a recent study, we developed an adaptive local validation procedure using a locus-based approach, which allowed us to successfully replicate a novel gene-gene interaction underlying high-density lipoprotein cholesterol (HDL-C) levels in multi-ethnic human cohorts [15]. The replicated gene-gene interactions were replicated in proximate, but different pairs of SNPs in the different ethnic populations, which can be due to either heterogeneity in LD patterns or real differences in the underlying causal interactions. In such scenarios [15], [38], a gene-based testing approach can prove powerful not only for the discovery of gene-gene interactions but also for their replication.

**Figure 1:**
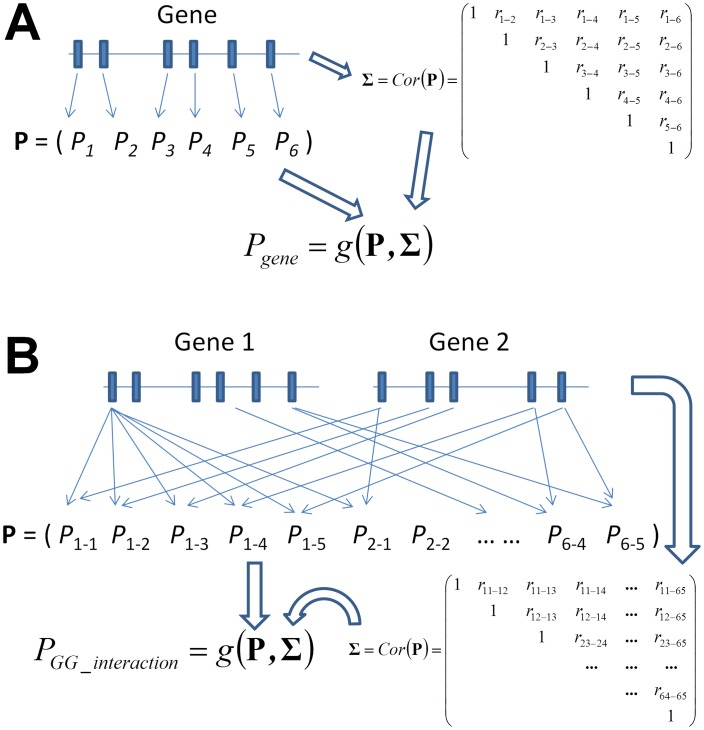
Graphical illustration of the framework of gene-based single-marker test and its generalization to a gene-based gene–gene interaction (GGG) test as proposed in this paper. While the former considers the *P* values of each single-marker test (A), a GGG test (B) is based on all *P* values of an interaction test between *each pair* of markers from each of the two genes. In order to combine these pairwise *P* values into a single test, a correlation matrix that concurrently accounts for linkage disequilibrium in each of the two genes needs to be estimated, which we derive in Materials and Methods.

Gene-based tests of main association effects can be classified into two categories, tests that consider multiple markers in a gene as part of a joint model [39], [40], [41], [42], [43], [44], [45], [46] and tests that combine marker-based test statistics or *P* values into a gene-based equivalent (Figure 1A) [31], [32]. One important advantage of the latter type of tests, which are the focus of this paper, is that they do not require any additional information once the marker-based interaction *P* values have been evaluated. While it is imperative to account for the correlation between tests of different markers that is due to LD, this can be achieved using estimates from an external reference panel if genotype information is not available. Here, we propose four gene-based gene-gene interaction (GGG) tests of quantitative traits by extending four existing methods of combining *P* values: (i) minimum p value [32], (ii) extended Simes procedure (GATES) [31], (iii) truncated tail strength [47], and (iv) truncated-product *P* value [48]. Our tests employ these methods to combine *P* values of interaction tests between all pairs of individual SNPs to obtain a *P* value for a GGG test, while accounting for the correlation between the individual *P* values (Figure 1B). A recent study has recently extended ATOM [41], a gene-based main effect test of the type that considers all markers in a gene in a joint model, to a gene-based test that collapses all markers in each gene prior to interaction testing [14]. An advantage of the *P* value combining approaches is that if there are multiple heterogeneous interactions between a pair of genes, first collapsing SNPs in each gene according to the former approach can average out these disparate signals and lead to a reduction in power. Other than *P* value combining approaches, linkage disequilibrium has often been utilized for detection of gene-gene interactions in case-control studies. By comparing LD patterns between cases and controls, Rajapakse et al. have recently developed a gene-based test of interactions for case-control studies [26].

Following the derivation of the statistical tests of GGG, we present extensive simulations with empirical LD patterns and allele frequencies that evaluate the type I error rates and power of these tests. They show all tests to have accurate type I error rates and to be more powerful than a test of the type that first considers a joint model of the markers in each gene, which we implement based on principal components [49], similarly to the aforementioned recently proposed method [14]. The simulations also suggest that the two truncated tests, which both go beyond considering the single strongest signal, are more powerful in cases when the interacting variants are not observed directly and might be partially tagged by different SNP-pairs and in cases of multiple causal interactions. We further present an empirical application of the novel methods, jointly with a curated human PPI network, to test for gene-level interactions underlying lipid levels in GWAS data from the Atherosclerosis Risk in Communities (ARIC) study [50]. We report five significant gene-level interactions associated with either total cholesterol (TC) or HDL-C levels, all of which are novel and are not significant when marker-based interaction tests are employed [15]. One of these suggestive gene-level interactions, between *SMAD3* and *NEDD9* on the levels of HDL-C, is significantly replicated in an independent cohort from the Multi-Ethnic Study of Atherosclerosis (MESA) [51].

## Materials and Methods

We test for interaction between two genes, each of which consisting of multiple SNP markers (Figure 1B). A “gene” in this context can be any locus or any collection of SNPs, with actual genes lending themselves to the test only due to underlying biology, not due to any statistical considerations. For a quantitative trait of interest, we apply a linear model approach to test for interactions between all pairs of SNPs between the two genes. We then describe a derivation of the correlation between these marker*-*based interaction test statistics, as well as a derivation that relies solely on external LD information, which should prove useful when genotype data for the individuals under study is not directly available. Accounting for the derived correlation, we extended four *P* value combining methods to combine those marker-based interaction *P* values into GGG *P* values (Figure 1B).

### Marker-based interaction test

The marker-based interaction test on which our gene-based approach is based is a standard linear model [6], [15]. Let 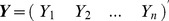 be the values of a quantitative trait of interest in a sample of *n* individuals, and let the genotype at two SNP markers be denoted as 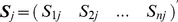 for *j* = 1, 2, with *S_ij_* (0, 1, or 2) being the number of copies of the reference allele at SNP *j* of individual *i*. The linear model with additive effects of the SNP-pair and their interaction can be written as,
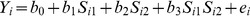
(1)where *b_i_* (*i* = 0, 1, 2, or 3) is the regression coefficient and *e_i_* is a residual that follows a normal distribution, N(0, 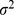). This model can be easily extended to include dominance effects and other interaction terms.[22] Using the matrix notation of 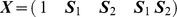, the least square estimates of the regression coefficients are 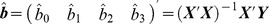, and the estimated variance-covariance matrix of 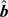 is 
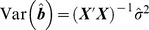
. The interaction between the two SNPs is then tested by testing the null hypothesis H_0_: 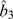 = 0, which leads to a *t*-test statistic, 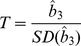 [15].

### Correlation between marker-based interaction test statistics

In the following, we derive the correlation between marker-based interaction tests which involve four SNPs, two in each of the two genes. First, suppose genotype data for these SNPs is available such that LD can be directly estimated. Let 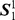 and 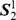 be the genotypes of the two SNPs in the first gene and 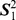 and 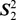 in the second gene, both in matrix notation. Let *T_ij_* denote the *t*-test statistic of the interaction between 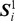 and 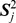. Our goal is to calculate the correlation between two interaction test statistics, which we refer here to the terms *T*_11_ and *T*_22_. While the case of the two tests having a SNP in common is a special case of this derivation in which the correlation between the two SNPs (the SNP and itself in that case) is 1, *T*_11_ and *T*_22_ are correlated due to LD between two SNPs in the same gene, for each of the two genes. We can calculate the correlation as,
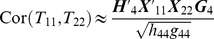
(2)where ***X***_11_ and ***X***_22_ are the two model matrices of the two interaction linear models as described in Equation (1), 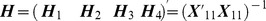, 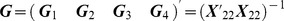, and *h*_44_ and *g*_44_ are the elements of ***H*** and ***G*** in the fourth row and the fourth column. The Supporting Text S1 describes a detailed derivation of Equation (2), which we also validated using simulations (Figure S1). We emphasize that the source of correlation is from correlation between different SNPs within the same genes, rather than correlation between the two genes, which are assumed to be in linkage equilibrium by the marker-based interaction test underlying our approach.

If genotype data for these SNPs is not available, correlation between pairs of SNPs can still be estimated, but only based on LD information from reference panels such as those from HapMap [52] or the 1000 Genomes Project [53]. In this case, we first derive the correlation between the two SNP products as
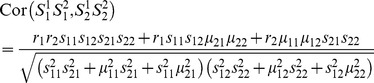
(3)where *r_i_* is the correlation coefficient between the two SNPs in the *i*th gene, and *μ_ij_* and *s_ij_* are the mean and standard deviation of *S_ij_* (refer to Supporting Text S1 for details). Based on this correlation between two SNP products, we then approximated the correlation between the two test statistics using a high-order polynomial estimated using simulations [31]. In cases when external LD information must be used, this polynomial (Figure S2) of Equation (3) should be used in place of Equation (2).

### Combining marker-based interaction *P* values into GGG *P* values

Between two genes with *m*_1_ and *m*_2_ SNPs, there are *m*_1_×*m*_2_ marker-based interaction *P* values, *p_ij_* (*i* = 1, …, *m*_1_; *j* = 1, …, *m*_2_). We can calculate the pairwise correlation matrix between these marker-based interaction test statistics, Σ, using Equation (2) or Equation (3), depending on whether genotype information is available. Using Σ, we are able extend four *P* value combining methods to four equivalent tests of GGG, GG_minP [32], GG_GATES [31], GG_tTS [47], and GG_tProd [48] as described in the following sections.

### GG_minP

The minimum *P* value is commonly used to combine *P* values of association tests of main effect in several programs, including PLINK [54] and VEGAS [32]. PLINK utilizes permutations to calculate a gene-based *P* value while accounting for the LD among SNPs, while VEGAS samples a large number of test statistics from given distributions and calculates a gene-based *P* value as the proportion of sampled minimum *P* values less than the observed minimum *P* value. Instead of using permutation or sampling, we adopt the method from Conneely and Boehnke [55] and integrate over a multivariate normal density function, MVN(0, Σ), to calculate a gene-based interaction *P* value,
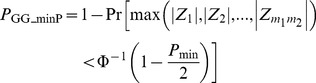
(4)where *Z_i_* (*i* = 1, …, *m*_1_ *m*_2_) follows a multivariate normal distribution MVN(0, Σ), Φ is the standard normal distribution function, and *P*_min_ is the minimum of the *m*_1_×*m*_2_ *P* values from the single marker-based tests. The GG_minP test of GGG is then defined as the two-sided test in Equation (4), which we implemented using the R package mvtnorm [56].

### GG_GATES

Liu et al. proposed a gene-based test of main effect, GATES, by extending Simes procedure to assess the gene level association significance [31]. GATES is similar to the minimum *P* value approach in that it picks the strongest signal in a gene, but is different in that the strongest signal does not have to be the one with the minimal *P* value as described in Equation (5). For *m*_1_×*m*_2_ ascending marker-based interaction *P* values, 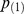, …, 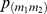, we define the GGG *P* value of GG_GATES as,
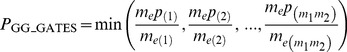
(5)where *m_e_* is the effective number of independent tests among the *m*_1_×*m*_2_ interaction tests and *m_e(j)_* is the effective number of independent tests among the top *j* interaction tests associated with the ordered *P* values, 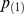, …, 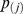. We estimate the effective number of tests, based on the correlations captured by Σ, using formulas derived by Moskvina and Schmidt [57].

### GG_tTS

While both GG_minP and GG_GATES only consider the strongest signal among the marker-based interaction *P* values to represent the gene level interaction, the tail strength method [58] combines signals from all marker-based *P* values. Jiang et al. extended the original tail strength method to a truncated version which only combines *P* values less than a predefined cutoff value, and demonstrated its superior power through simulations [47]. We derived the GG_tTS statistic for GGG as,
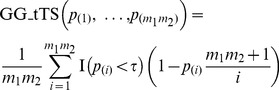
(6)where I(.) is an indicator function and *τ* is a predefined cutoff value of which *P* values are to be combined. Throughout this study, we set *τ* to 0.05 (nominal significance level), as recommended in Zaykin et al. [48]. Intuitively, GG_tTS weighs all the *P* values that pass the cutoff of *τ*, with the last term in Equation (6) denoting the weights, and becomes larger the smaller the *P* values. Since the marker-based interaction *P* values are correlated due to LD between SNPs in a gene, the null distribution of GG_tTS is unknown. We calculate empirical *P* values for GG_tTS using a similar sampling approach to that described in Zaykin et al. [48] and Liu et al. [32]. First, we repeatedly simulate the interaction test statistics from a multivariate normal distribution with correlation calculated from Equation (2) using mvtnorm [56] and calculate the GG_tTS statistic for each simulation. Then we calculate the empirical *P* value as the proportion of simulations for which the GG_tTS estimate is larger than the observed one.

### GG_tProd

Similar to GG_tTS, we define a GGG test statistic for the GG_tProd method [48] by a product function of the marker-based interaction *P* values which are less than a cutoff value, *τ*,
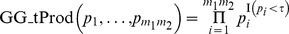
(7)


As the marker-based interaction tests between two genes are correlated, there is no analytic solution for the distribution of the two test statistics described in Equation (7). Thus, empirical *P* values for GG_tProd are calculated using a similar approach described above.

### Gene-based interaction test using principal components (GG_PC)

Principal components (PC) have been used to aggregate information in a gene-based test of main association effect [40]. We included a PC-based method [14] in our study for comparison purposes. The approach identifies PCs accounting for 90% of the variance for each gene and then performs a global test for interaction between PCs in a linear model framework similar to Equation (1) with multiple pairwise interaction terms between PCs of the two genes [14]. In the case where there are *L*_1_ PCs in gene 1 and *L*_2_ PCs in gene 2, there will be *L*_1_×*L*_2_ interaction terms in the linear model. The interaction was tested through an *F*-test with *L*_1_×*L*_2_ degrees of freedom comparing two models with and without interaction terms. Importantly, both GG_PC as used here and all other GGG tests included in this study test for pure interaction effects, that is on top and beyond any marginal effects, which is achieved by testing the null hypothesis that the interaction term is zero.

### Simulation studies of type I error rate and power

To evaluate the performance of our gene-based interaction tests using data with realistic LD patterns, we picked two loci in linkage equilibrium from the imputed genotype data of ∼10,000 European American samples in the ARIC study [15], [50]. The first locus contains 53 SNPs from which 14 tag SNPs were selected using Haploview [59]. The second locus contains 28 SNPs including 10 tag SNPs. The LD patterns of the two loci and tag SNPs are shown in Figure S3.

In each simulation, a random sample of size *n* was drawn without replacement from the population of ∼10,000 EAs. We simulated both scenarios where causal variants are observed or not (to consider scenarios in which they are not genotyped) by only testing interactions between tag SNPs [14], which may or may not include causal variants. For the PC-based method, we utilized the PCs of the tag SNPs in the two genes. GGG tests combine *P* values across all pairs of tag SNPs into gene-based interaction *P* values. When calculating the correlation between marker-based interaction test statistics, we used Equation (2) or (3), depending on the simulated scenarios where individual genotype data are accessible (Equation 2) or not (Equation 3).

To evaluate the type I error rate, we simulated the phenotype as a random error which follows a standard normal distribution. We varied the sample size *n* and the nominal significance level (Table 1). For power evaluation, we simulated the phenotype as the sum of the genotypic values of the causal SNP-pairs, their interaction, and a random error which follows a standard normal distribution, as described in Equation 1. We varied sample size *n*, number of causal SNP-pairs, effect size of the causal interaction, and minor allele frequency. We also simulated three scenarios where the actual interaction occurs between unobserved SNPs (U-U), between unobserved and observed SNPs (U-O), and between observed SNPs (O-O). Here the observed SNPs refer to the tag SNPs. Both type I error rates and power were estimated by the proportions of simulations that resulted in significant *P* values out of 10,000 and 5000 simulations, respectively.

**Table 1:** Empirical, simulation-based type I error rates of proposed GGG tests.

*n*	α	GG_PC	GG_minP	GG_GATES	GG_tTS	GG_tProd
1000	0.05	0.0506	0.0502	0.0564	0.0521	0.0492
	0.01	0.0101	0.0099	0.0105	0.0113	0.0094
2000	0.05	0.0496	0.0474	0.0531	0.0508	0.0452
	0.01	0.0092	0.0087	0.0091	0.0117	0.0088
3000	0.05	0.0504	0.0493	0.0557	0.0489	0.0528
	0.01	0.0087	0.0082	0.0088	0.0099	0.0120
5000	0.05	0.0506	0.0485	0.0564	0.0511	0.0495
	0.01	0.0103	0.0086	0.0090	0.0096	0.0098

### Application with protein–protein interactions (PPI) to GWAS

All work done in this paper was approved by local institutional review boards or equivalent committees.

We obtained Affymetrix 6.0 SNP array genotypes of 9,713 European American samples from the ARIC study [50]. The genotype data were further imputed to ∼2.5 million SNPs using MACH [60]. We considered four lipid measurements: total cholesterol (TC), HDL-C, low-density lipoprotein cholesterol (LDL-C), and triglyceride (TG). All measurements were done in the fasting state using standard enzymatic methods. Each lipid level is measured at multiple time points and we considered the average level per individual of each lipid in all our analyses [61]. We applied a log transformation to TG levels to normalize them because of the skewness in the original distribution [61]. We excluded individuals known to be taking lipid-lowering medications. Gender, age, age squared, and body mass index (BMI) were included as covariates in all analyses [61], [62], [63]. Similar to the four lipid phenotypes, we considered average values for age and BMI whenever multiple measurements were available. Principal component analysis was conducted using EIGENSOFT [64], and top 10 PCs were included in the analysis as covariates to account for potential population stratification.

We assembled 2,974 high-confidence human PPIs [15] and for each pair of interacting proteins exhaustively tested the pairwise interactions between each SNP in the first gene and each SNP in the second gene. We obtained gene information (hg18) from UCSC genome browser to map SNPs to genes, and considered all SNPs between 5 kb upstream and 5 kb downstream of the gene. For *n*_1_ and *n*_2_ being the numbers of SNPs in the first and second gene, respectively, the number of marker-based interaction tests is *n*_1_×*n*_2_ for this PPI. As a result, the marker-based interaction analyses failed to identify any significant interactions associated with the four lipid levels after multiple-testing correction [15]. We then applied the four GGG tests, GG_minP, GG_GATES, GG_tTS, and GG_tProd to combine these *n*_1_×*n*_2_ marker-based *P* values to a GGG *P* value for each PPI. We note that a physical protein-protein interaction does not necessarily entail a statistical gene-gene interaction underlying the studied trait, or vice versa, but by focusing on pairs of genes whose proteins interact, we aim to increase the likelihood of a pair of tested genes to exhibit a gene-gene interaction, thereby increasing the power of detection and replication of such interactions.

For computational efficiency and robustness, we adopted an upper limit of 500 marker-based interaction *P* values to be combined into a gene-based *P* value. Therefore, large gene pairs which have more than 500 marker-based interaction *P* values were divided into subgroups containing 500 *P* values or less and each subgroup was combined into a GGG test. In order to further improve the efficiency for GG_tTS and GG_tProd, we used an adaptive sampling procedure when calculating empirical *P* values. This adaptive procedure included the following three steps. First, we sampled 1000 random vectors from the target distribution and calculated the empirical *P* value. If the empirical *P* value is less than 0.01, then we perform additional 99,000 samplings. If the updated empirical *P* value is less than 1×10^−4^, we do additional 99,900,000 samplings. As a result, the maximum number of simulations is 10^8^ in this adaptive procedure and the minimal possible empirical *P* value is 1×10^−8^, which is below the multiple-testing corrected threshold, 10^−6^, in this study. The number of samplings in each of the three steps can be modified according to the required significance level after correction for multiple testing.

## Results

### Type I error rate

We first set out to verify the type I error rates of the five gene-based interaction tests, GG_minP, GG_GATES, GG_tTS, GG_tProd, and GG_PC. To estimate these, we considered randomly simulated phenotypes with real genotype data, thereby maintaining empirically observed LD patterns and minor allele frequencies (Materials and Methods). In each simulation, a random sample of *n* individuals was drawn and interaction was tested between two loci using tag SNPs alone (14 and 10 tag SNPs in each locus respectively). We varied *n* from 1000 to 5000 and considered two nominal significance levels, 0.01 and 0.05. For each parameter setting, we evaluated the type I error rate from 10,000 simulations. All five GGG tests have type I errors consistent with the nominal significance level (Table 1). To ensure the type I error rates were not affected by the number of interactions combined into a GGG test, we conducted another set of simulations using more SNPs (30 and 20 randomly selected SNPs in each locus respectively) and still observed type I error rates consistent with the nominal significance levels (Table S1).

### Statistical power

To evaluate the statistical power of the five GGG tests, we repeated simulations with empirically observed LD patterns with random pair or pairs of SNPs selected to exhibit interaction. We define the level of the quantitative trait in the simulations to be the sum of the genotypic values of the causal SNP-pair/s, their interaction, and a random error. Gene-based interaction tests were applied as above, based on tag SNPs, while each causal interaction was simulated in one of three scenarios, with none (U-U), one (U-O), or both (O-O) SNPs observed as tag SNPs. As expected, power of all tests is affected greatly by the sample size, *e.g.* for the case of two unobserved interacting SNPs (U-U), the power of the different tests ranges between 14–47% for *n = *1000, while it ranges between 73–99% for *n = *5,000 (Table 2). It also depends on the effect size of the interaction, with a difference, when the interacting SNPs are directly observed (i.e. directly tested; O-O), between effect size of 0.15 to 0.25 at least doubling the power for a given sample size of *n = *1000 (Table 2). Minor allele frequencies (MAF) of the interacting SNPs have a considerable effect on power as well, *e.g.* because the 29th SNP in locus 1 has a relatively low MAF of 0.1, all tests have lower power estimates for the interaction of SNP-pair “29-17” compared to other SNP-pairs (Table 2). The number of interacting pairs of SNPs is another factor contributing to power, as is whether the causal SNP-pairs are observed or not (Table 2).

**Table 2:** Empirical, simulation-based statistical power of GGG tests.

Simulation number	Interacting SNP-pairs^a^	Type^b^	MAFs^c^	Effect size^d^	*n*	Power^e^				
						GG_PC	GG_minP	GG_GATES	GG_tTS	GG_tProd
1	30-15	U-U	.45-.48	0.15	1k	14.3	31.0	34.8	47.2	47.0
					2k	27.5	60.0	65.2	76.3	76.0
					3k	43.9	81.8	84.4	90.6	90.6
					5k	73.3	94.5	98.0	98.3	99.3
2	30-17	U-O	.45-.39	0.15	1k	14.1	30.4	33.6	45.5	45.4
					2k	27.1	60.9	64.5	73.5	73.4
					3k	44.5	83.6	85.1	88.9	89.0
					5k	75.1	93.4	98.2	98.2	98.9
3	29-17	O-O	.10-.39	0.15	1k	7.0	10.2	11.0	8.4	8.5
					2k	10.9	16.8	19.4	13.6	14.3
					3k	14.2	28.6	30.4	20.5	21.2
					5k	21.5	51.4	52.9	33.7	35.0
4	29-17	O-O	.10-.39	0.25	1k	13.1	25.3	27.0	18.4	19.1
					2k	27.6	56.0	57.9	35.7	38.8
					3k	40.8	81.4	82.6	52.8	60.4
					5k	69.3	97.7	98.0	75.8	85.9
5	30-15, 40-20, 48-27	U-U	.45-.48, .41-.34, .30-.43	0.12	1k	17.8	33.5	37.0	49.3	49.1
					2k	38.8	64.3	69.3	80.1	79.9
					3k	57.7	83.9	86.5	92.2	92.0
					5k	87.2	95.3	97.9	99.4	98.5
6	30-15, 40-20, 48-27	U-U	.45-.48, .41-.34, .30-.43	0.15	1k	28.3	51.5	55.8	68.1	68.2
					2k	60.5	85.8	88.2	94.3	94.3
					3k	83.7	97.4	98.2	99.4	99.4
					5k	98.7	99.9	99.9	100	100
7	29-17, 39-22, 47-25	O-O	.10-.39, .41-.38, .29-.44	0.12	1k	18.8	42.8	47.1	54.3	54.3
					2k	39.3	77.5	81.0	84.6	84.7
					3k	57.5	93.7	94.5	95.9	95.7
					5k	88.3	98.8	99.9	99.9	100
8	10-5, 20-10, 30-15, 40-20, 48-27	U-U	.10-.39, .10-.44, .45-.48, .41-.34, .30-.43	0.12	1k	23.1	34.8	38.1	43.6	43.5
					2k	51.1	67.5	71.8	79.0	79.0
					3k	73.7	86.2	89.1	93.0	93.0
					5k	96.4	96.6	98.7	99.2	99.7
9	6-4, 19-9, 29-17,39-22, 47-25	O-O	.12-.49, .32-.47, .10-.39, .41-.38, .29-.44	0.12	1k	54.9	89.7	92.1	96.3	96.3
					2k	92.4	99.7	99.8	100	100

a Indices of the interaction SNPs in the two loci (Figure S3); Three types of scenarios are considered, of one, three, and five pairs of interacting SNPs, for each at least two different sets of SNPs are considered, for a total of 7 different scenarios.

b U: SNP untyped; O: SNP observed; For scenarios with more than one pair of interacting SNPs, the U/O status of the first and second interacting SNP is the same across all pairs.

c Minor allele frequency of all SNPs involved in interactions, by order.

d Coefficient of the interaction term in the linear model, *b*_3_, as described in Equation (1); For scenarios with more than one pair of interacting SNPs, the effect size is the same for all pairs.

e Power, as percentage of significant tests with *P* value<0.05.

**Figure 2:**
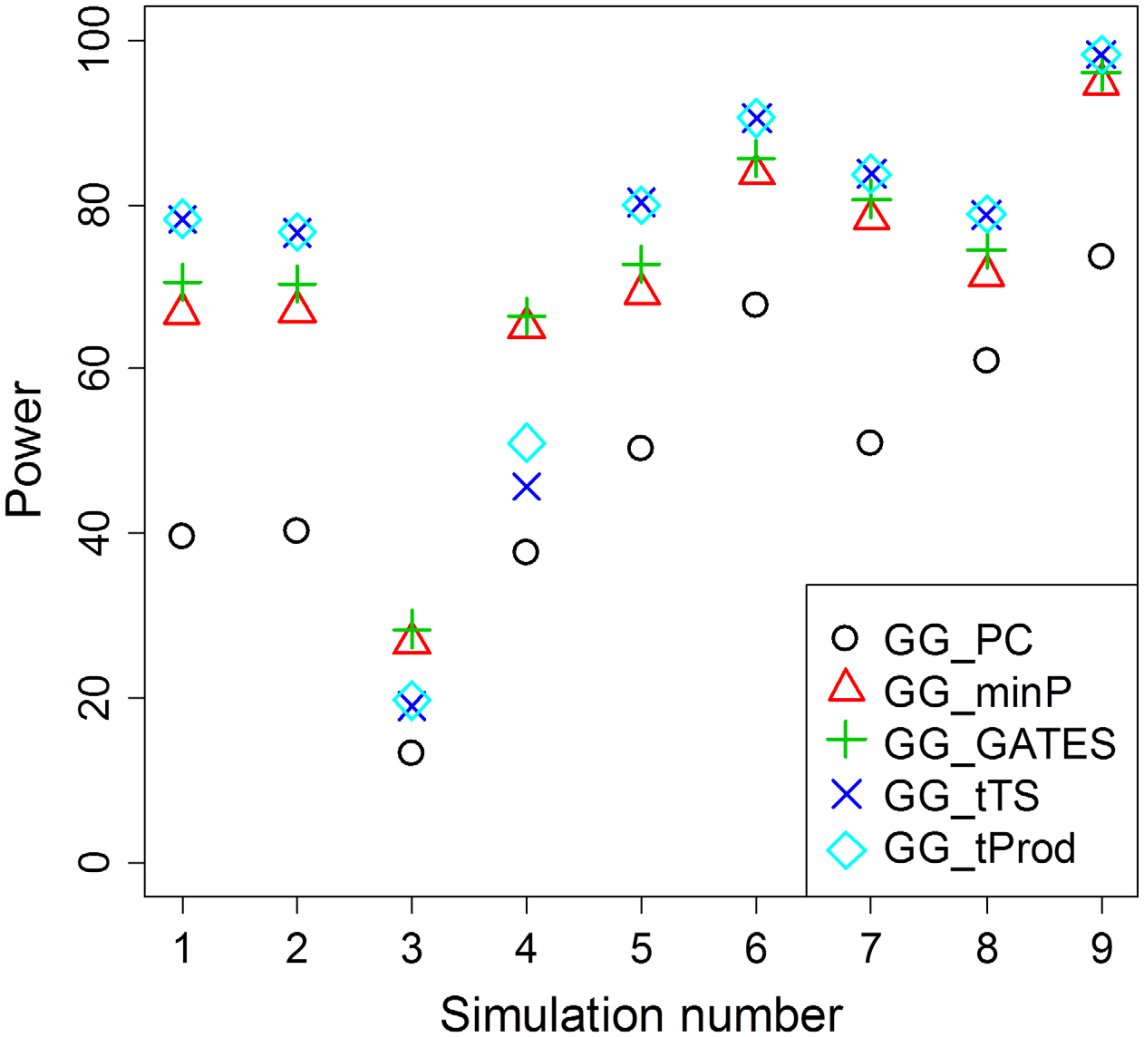
Average power of GGG tests summarized from Table 2. For each simulation scenario from Table 2, average power for each type of test is presented as an average across the different sample sizes (*n*) reported in Table 2. The method that collapses markers in each of the two genes, GG_PC, is least powerful in all simulation scenarios. Among the four GGG tests that combine *P* values, GG_minP and GG_GATES are more powerful only in simulation scenarios 3 and 4, which are the only cases that we simulated a single marker-by-marker interaction with both markers available for analysis (denoted by O-O in Table 2). GG_tTS and GG_tProd are most powerful in all other simulation scenarios.

In all simulated scenarios, GG_PC, which takes the approach of first collapsing markers in each of the two genes, is less powerful than the four *P* value combining GGG tests (Table 2; Figure 2), which may be due to a combination of the principal components not fully capturing the underlying interaction signals and the multiple degrees of freedom associated with that test statistic. As both GG_minP and GG_GATES consider the best signal to represent a gene level interaction, they exhibit very similar levels of power, although GG_GATES is slightly more powerful in all simulated scenarios (Table 2; Figure 2). While GG_minP picks the smallest *P* value to represent a gene-level interaction, GG_GATES picks the strongest signal while accounting for the effective number of tests, which may not necessarily be the smallest *P* value, which explains the gain in power.

GG_tTS and GG_tProd both combine evidence from all marker-based interaction *P* values below a pre-determined threshold (Materials and Methods). These two tests show very similar levels of statistical power, with any small differences in power being attributable to the shape of the tail of the distribution of *P* values (Figure 2). The main difference between the two tests is that GG_tTS differentially weights the ordered P values before combining them. Comparing the power of GGG tests that consider only the single strongest signal (GG_minP and GG_GATES) with tests that combine several relatively significant signals (GG_tTS and GG_tProd), in almost all scenarios the latter exhibit superior power (Table 2; Figure 2). An exception is the case of a single pair of interacting SNPs that are directly observed and available for testing. In this case, GG_minP and GG_GATES exhibit considerably superior power across all simulated effect sizes and sample sizes (Table 2; Figure 2). In all other scenarios, namely when either or both of the pair of interacting SNPs is/are not directly observed, or when multiple pairs of (observed or unobserved) SNPs are interacting, the strategy of aggregating the significance signal across multiple pairs of SNPs, as implemented in GG_tTS and GG_tProd, has the upper hand (Table 2; Figure 2). For the case of multiple interactions, it is clearly expected that GG_tTS and GG_tProd yield better results as they aggregate these independent signals [31], [41]. For the cases where at least one of the interacting SNPs is not directly observed, the increase in power likely stems from multiple observed SNPs (in LD with the unobserved contributing SNP/s) jointly capturing the signal better than any individual observed SNP.

Noticing that power is generally low for all GGG tests when the MAFs of the causal variants are lower (Table 2), we performed an additional set of simulations with yet lower frequency variants (Table S2). We observed limited power for lower frequency variants, though a similar pattern emerged that GG_tTS and GG_tProd are usually more powerful than other tests, while GG_minP and GG_GATES are more powerful only when there is a single, and directly observed causal interaction (Table S2).

### Robustness with external LD

Four of the GGG tests use LD information for estimating the correlation between tests for different pairs of SNPs (Equation 2). When genotyping or sequencing data are available for each individual, these can be readily estimated, which is the situation we considered thus far. However, we also aim for these tests to be applicable to situations in which *P* values for each pair of SNPs are available, but not the actual genotyping or sequencing data. In such cases, external LD information from data of proximate ethnicity can be used as a proxy for LD in the data by evaluating the covariance between tests via Equation 3 (Materials and Methods). We examined type I error rates and power in this scenario, where LD information was estimated from a combined panel of two population of European ancestry (CEU+TSI) in data from HapMap3 [52], [65]. The type I error rate is still consistent with the nominal significance level in this scenario when using Equation 3 (Table S3). Power is lower, but only slightly, compared to when individual genotyping data are available as in the previous set of simulations above (Table S4).

### Application with PPI to GWAS on lipid levels

We applied all GGG tests (except GG_PC, due to its limited power) to real quantitative trait data from 9,713 European American individuals from the ARIC study. We considered for analysis the levels of four lipids: TC, HDL-C, LDL-C and TG. For each, we tested for gene-based interaction between each pair of genes based on 2,974 high-confidence human PPIs. We further divided gene pairs that have more than 500 SNP-pairs into loci that we analyzed separately (Materials and Methods), resulting in 12,320–13,254 gene-based (or locus-based) interaction tests for each lipid level. In total, *P* values for a total of ∼6 million pairs of SNPs were obtained and combined to gene-based statistics of the four types. The conservative genome-wide significance level for our gene-based tests after Bonferroni correction is about 9.4×10^−7^ (α = 0.05 divided by at most 13,254 gene-based tests and divided by 4 traits tests). The Bonferroni corrected significance level if each pair of SNPs in each PPI was tested separately using a marker-based test would have been much lower, 2×10^−9^. Our recent study has detected no significant SNP-by-SNP interactions at that significance level based on the same PPIs [15].

The GG_tTS test detected 5 significant gene-level interactions, underlying TC and HDL-C, with *P*<9.4×10^−7^ (Table 3). The GG_ tProd test detected 1 significant gene-level interaction, which is one of the 5 detected by GG_tTS (Table 3). While our simulations use equal effect sizes for all causal interactions, if they are different in the particular application to real data, it can explain the differences in *P* values of the two tests (Table 3). The GGG tests based on the single strongest signal alone (GG_minP and GG_GATES) produced no significant results. These results point to the importance of combining different signals across a pair of genes (GG_tTS and GG_tProd) relative to both marker-based tests based on pairs of individual SNPs [15] and GGG tests based on only a single strongest signal (GG_minP and GG_GATES). Also considering the potential differences in the effect sizes of the underlying causal interactions, GG_tTS can be a better choice than GG_tProd in real data analysis. Combined with the simulation results (Table 2), these results suggest that the causal interaction is either more complex than a single SNP-by-SNP interaction or that the causal SNPs are not completely tagged in these imputed data of 2.5 million SNPs.

**Table 3:** Significant (*P*<9.4×10^−7^; bolded) gene-level interactions affecting total cholesterol (TC) and high-density lipoprotein cholesterol (HDL-C) levels in data from the ARIC study.

Trait	Gene 1	Gene 2	*P* values			
			GG_minP	GG_GATES	GG_tTS	GG_tProd
TC	*HDAC2*	*HDAC1*	1.8×10^−2^	2.3×10^−3^	**1.0×10^−7^**	1.9×10^−4^
	*APP*	*APBB2*	6.5×10^−2^	3.9×10^−3^	**6.5×10^−7^**	3.5×10^−6^
HDL-C	*SMAD3* *	*NEDD9* *	2.5×10^−2^	1.2×10^−2^	**8.4×10^−7^**	**5.0×10^−7^**
	*RPS6KA2*	*MAPK1*	6.2×10^−3^	8.2×10^−4^	**2.6×10^−7^**	2.2×10^−4^
	*KDM4A*	*HIST1H3F*	3.7×10^−3^	2.2×10^−4^	**1.3×10^−7^**	2.1×10^−6^

* The interaction between *SMAD3* and *NEDD9* on HDL-C levels was further replicated in data from the MESA study (multiple testing corrected *P*_c_ = 0.01 for GG_tProd and *P*_c_ = 0.05 for GG_tTS).

Using 2,685 European American samples from MESA, we successfully replicated the gene-level interaction that was supported by both GG_tTS and GG_tProd, between *SMAD3* and *NEDD9*, on HDL-C levels. Replication is significant after correcting for the 5 gene-level interactions of interest using both GG_tProd (multiple testing corrected *P*_c_ = 0.01) and GG_tTS (*P*_c_ = 0.05). The other four interactions did not significantly replicate. *SMAD3* is a transcriptional modulator activated by transforming growth factor β (TGF-β) [66], [67] and has been reported to be marginally associated with coronary artery disease, of which low HDL-C levels is a risk factor [68]. *NEDD9* has been associated with Alzheimer's disease [69], [70], which has been recently claimed to share genetic risk factors with cholesterol levels [71]. Neither of the two genes has been previously associated with lipid levels. To examine this further, we tested for main (marginal) associations of all SNPs in the ten genes involved in gene-based interactions (Table 3) and found none to be significantly associated by itself with any lipid level following multiple-testing correction (Figure S4). We also performed gene-based tests of main effects for the ten genes on four lipid levels, but found no gene-level marginal associations (Table S5).

## Discussion

This study proposed GGG tests that combine marker-based interaction tests into a single *P* value of a gene-by-gene interaction underlying quantitative traits. These can be viewed as an extension of similar approaches that have proven successful for detecting main effects in GWAS [28]. What made the extension possible is the derivation of the correlation structure of the marker-based interaction tests that is due to LD in each of the two genes, which our tests allow incorporating either directly or based on LD from an external reference panel. All four proposed GGG tests, GG_minP, GG_GATES, GG_tTS, and GG_tProd, have correct type I error rates, and are more powerful than a GGG test that collapses each gene to its principal components, GG_PC. As expected, GG_GATES and GG_minP, which are based on testing the single most extreme signal, are more powerful in the simple case of a single and fully-observed interaction. Among those four tests, GG_tTS and GG_tProd are more powerful in cases where there are multiple causal interactions as they aggregate multiple signals into a single gene-level signal. Even in the case of a single causal interaction, if one or both causal variants are not directly observed, GG_tTS and GG_tProd still provide an improvement in power, presumably due to aggregating signals from different SNP-pairs that are each only partially linked to the causal SNP-pair. When applied to real data, GG_tTS shows better power than GG_tProd by having smaller P-values for four out of the five interactions shown in Table 3. The proposed tests can potentially be extended both to more complex types of interaction effects and to dichotomous, case-control data using a similar *P* value combining framework. The major modification needed for dichotomous traits is a new derivation of the correlation between the marker-based interaction test statistics in a logistic regression model.

The computational burden for a GGG analysis is minimal once marker-based interaction *P* values have been obtained. Both GG_minP and GG_GATES are fast as they do not require any sampling from empirical distribution or permutations. The other two tests, on the other hand, estimate empirical *P* values by sampling a large number of random vectors that follow a multivariate normal distribution dictated by the estimated parameters. The computational burden can be reduced using several procedures such as the adaptive procedure that we applied of first sampling a small number of vectors and only increasing the sample size when the empirical *P* value is small [72]. In practice, sampling a large number of vectors is only required for a few highly significant interactions. Another way to speed up the analysis is to apply the tests that aggregate multiple signals, GG_tTS and GG_tProd, only in cases where the more efficient GG_minP or GG_GATES points to *P* values below a certain threshold. Our results suggesting much improved power of GG_tTS and GG_tProd in certain scenarios entail that this initial threshold should not be too strict, e.g. it can be one of nominal significance, without fully correcting for multiple testing.

When marker-based interaction *P* values are available, the proposed gene-based tests can be used even without individual-level data. This makes the tests readily applicable to an enormous amount of publicly available data that could be re-analyzed using this approach. Moving from the marker level to the gene level makes a genome-wide interaction analysis, with sample sizes as observed in GWAS, more promising since the multiple hypothesis testing burden becomes orders of magnitude smaller. The GGG tests proposed here can be applied to all pairs of genes genome-wide. Alternatively, to allow further reduction in both multiple testing burden and computing time, they can be applied to a focused subset of pairs of genes that is likely to be enriched for gene-gene interactions. Such a subset can be, for instance, all pairs of genes that are known to be involved in protein-protein interactions [73], [74] or other type of physical interaction [74], or pairs of genes that share a function [75] or play a part in the same pathway/s [76]. A more enriched set can potentially be obtained by further focusing on sets of genes based on knowledge specific to the studied trait, e.g. based on known associations of this and similar traits, gene ontology, or participation in pathways relating to the trait. Finally, we note that the units of testing do not necessarily have to be a physical gene, but can rather be any loci of interest.

We applied the proposed methods to test for gene-level interactions underlying lipid levels. As an enriched set of gene-pairs, we considered all pairs of genes where the corresponding proteins exhibit an interaction according to a high-confidence human PPI network (without further knowledge specific to the studied traits). We discovered five gene-level interactions underlying lipid levels that approach significance. All the interactions appear to be more complex than expected from a single SNP-by-SNP interaction, which is likely the reason none were detected in our recent marker-based study of the same data [15]. One of the five gene-level interactions, between *SMAD3* and *NEDD9* in their effect on HDL-C levels, was further replicated in an independent European American cohort. While a statistical gene-gene interaction does not necessarily entail an epistatic interaction, it is interesting to note that the *TGF-b*/*Smad3* signaling pathway has an important role in regulating glucose and energy homeostasis and that Smad3-deficient mice are protected from diet-induced obesity and diabetes [77]. *NEDD9* (neural precursor cell expressed, developmentally down-regulated 9) has been associated with the risk of developing Parkinson's disease and late-onset Alzheimer's disease, a disorder whose pathogenesis is modulated by cholesterol levels and cholesterol-related genes [69], [70], [78].

## Supporting Information

Figure S1 Scatter plot of empirical correlation using simulation and analytical correlation calculated by Equation (2).
(DOC)

Figure S2 Scatter plot of correlation between interaction test statistics and correlation between products of SNP pairs by Equation (3). The red line is the estimated fifth degree polynomial (y = 0.33181x−2.50443x^2^+10.21850x^3^−11.09725x^4^+4.05560x^5^), which is applied when external LD information is used. The R square value of the polynomial model is 0.986.
(DOC)

Figure S3 LD patterns of two empirical loci used in simulation studies. Figures are LD plots produced using Haploview [59]. The 14 and 10 tag SNPs in locus 1 (a) and locus 2 (b), respectively, are denoted by blue squares. These tag SNPs alone were considered for interaction testing.
(DOC)

Figure S4 QQ-plots of marginal association testing of SNPs from the 10 genes from Table 3 for four lipid levels.
(DOC)

Table S1 Empirical, simulation-based type I error rates of GGG tests using more SNPs (30 and 20) in the two genes compared to Table 1 in main text.
(DOC)

Table S2 Empirical, simulation-based statistical power of GGG tests (in percentage) for low-frequency variants.
(DOC)

Table S3 Empirical, simulation-based type I error rates of GGG tests using external LD information.
(DOC)

Table S4 Empirical, simulation-based statistical power of GGG tests (in percentage) using external LD information. Except for the test being based on external LD information (Equation 3 instead of Equation 2), the table mirrors Table 2 in the main text.
(DOC)

Table S5 Results of gene-based tests of marginal associations of the ten genes (Table 3) on four lipid levels in ARIC.
(DOC)

Text S1 Derivations of Equations (2) and (3).
(DOC)
