# Supplementary material for: Gene-Based Testing of Interactions in Association Studies of Quantitative Traits
Source: PLoS Genet. 2013 Feb 28;9(2):e1003321. doi: 10.1371/journal.pgen.1003321 (PMC3585009; doi:10.1371/journal.pgen.1003321)

**Figure S1. Scatter plot of empirical correlation using simulation and analytical correlation calculated by Equation (2).**


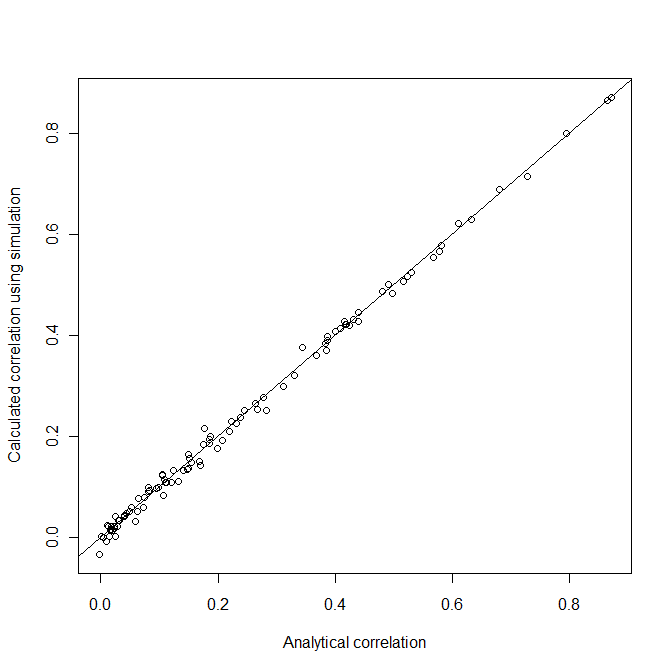

Supplement: Figure S1 — Scatter plot of empirical correlation using simulation and analytical correlation calculated by Equation (2). (DOC) [file pgen.1003321.s001.doc]
