# Supplementary material for: Gene-Based Testing of Interactions in Association Studies of Quantitative Traits
Source: PLoS Genet. 2013 Feb 28;9(2):e1003321. doi: 10.1371/journal.pgen.1003321 (PMC3585009; doi:10.1371/journal.pgen.1003321)

**Figure S2. Scatter plot of correlation between interaction test statistics and correlation between products of SNP pairs by Equation (3).** The red line is the estimated fifth degree polynomial (y = 0.33181x - 2.50443x^2^ + 10.21850x^3^ - 11.09725x^4^ + 4.05560x^5^), which is applied when external LD information is used. The R square value of the polynomial model is 0.986.


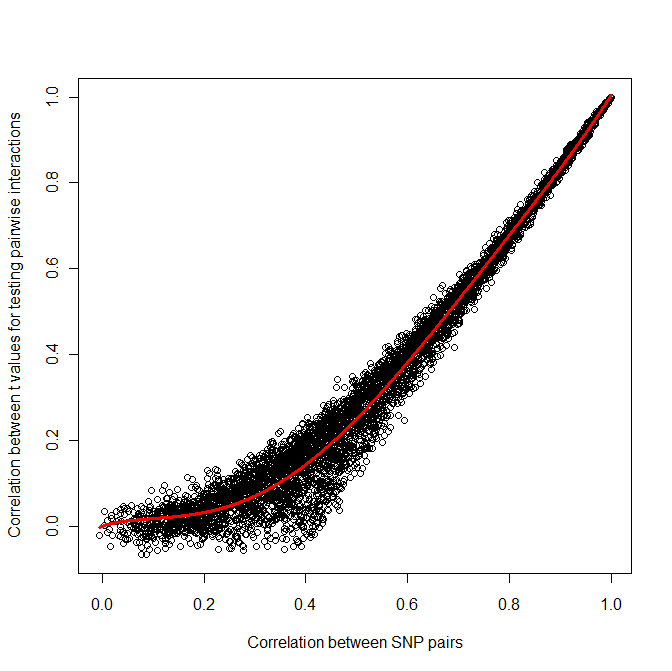

Supplement: Figure S2 — Scatter plot of correlation between interaction test statistics and correlation between products of SNP pairs by Equation (3). The red line is the estimated fifth degree polynomial (y = 0.33181x−2.50443x2+10.21850x3−11.09725x4+4.05560x5), which is applied when external LD information is used. The R square value of the polynomial model is 0.986. (DOC) [file pgen.1003321.s002.doc]
