# Supplementary material for: Gene-Based Testing of Interactions in Association Studies of Quantitative Traits
Source: PLoS Genet. 2013 Feb 28;9(2):e1003321. doi: 10.1371/journal.pgen.1003321 (PMC3585009; doi:10.1371/journal.pgen.1003321)
Supplement: Figure S3 — LD patterns of two empirical loci used in simulation studies. Figures are LD plots produced using Haploview [59]. The 14 and 10 tag SNPs in locus 1 (a) and locus 2 (b), respectively, are denoted by blue squares. These tag SNPs alone were considered for interaction testing. (DOC) [file pgen.1003321.s003.doc]

**Figure S3. LD patterns of two empirical loci used in simulation studies.** Figures are LD plots produced using Haploview . The 14 and 10 tag SNPs in locus 1 (**a**) and locus 2 (**b**), respectively, are denoted by blue squares. These tag SNPs alone were considered for interaction testing.

**a**


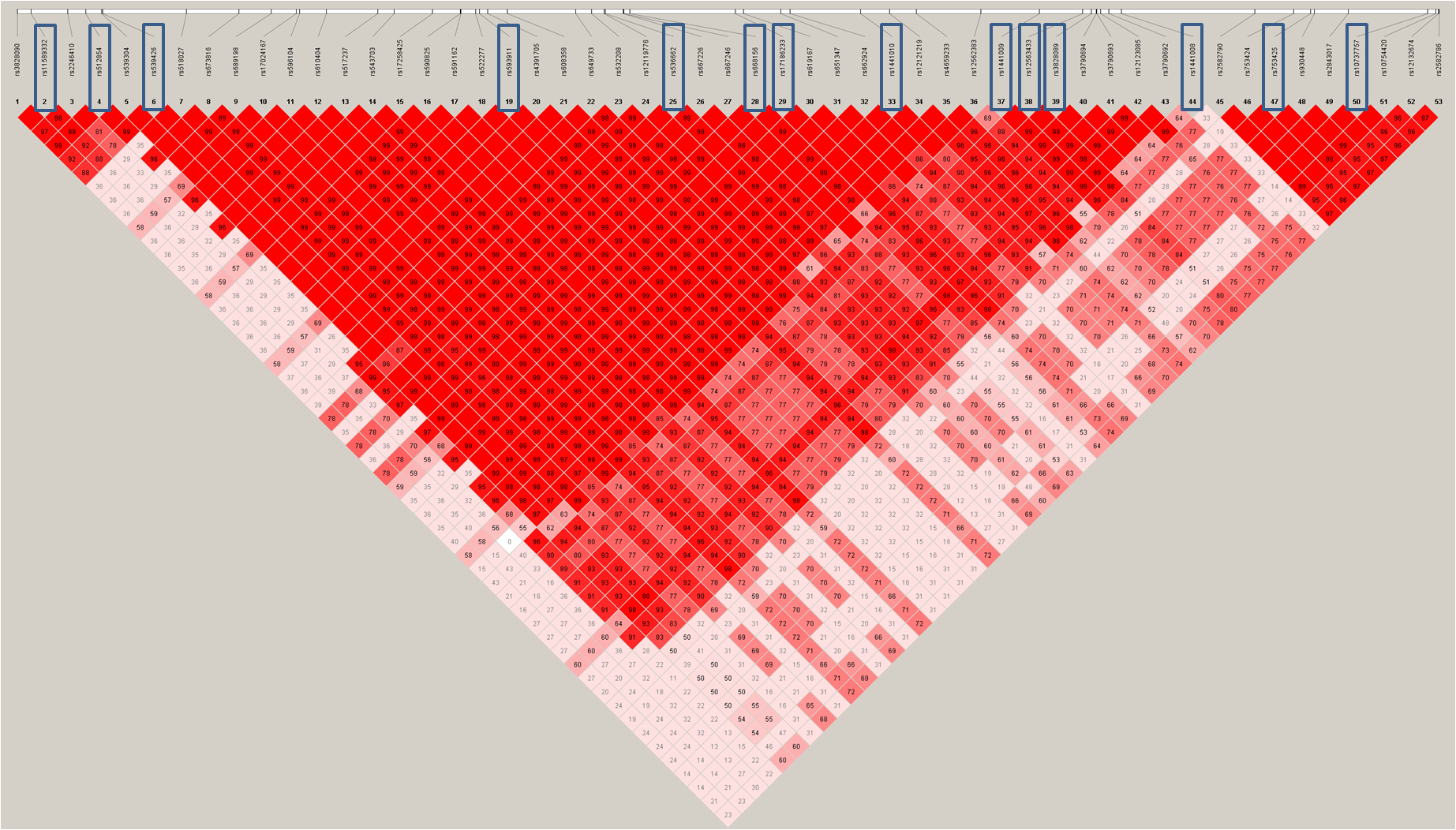


**b**


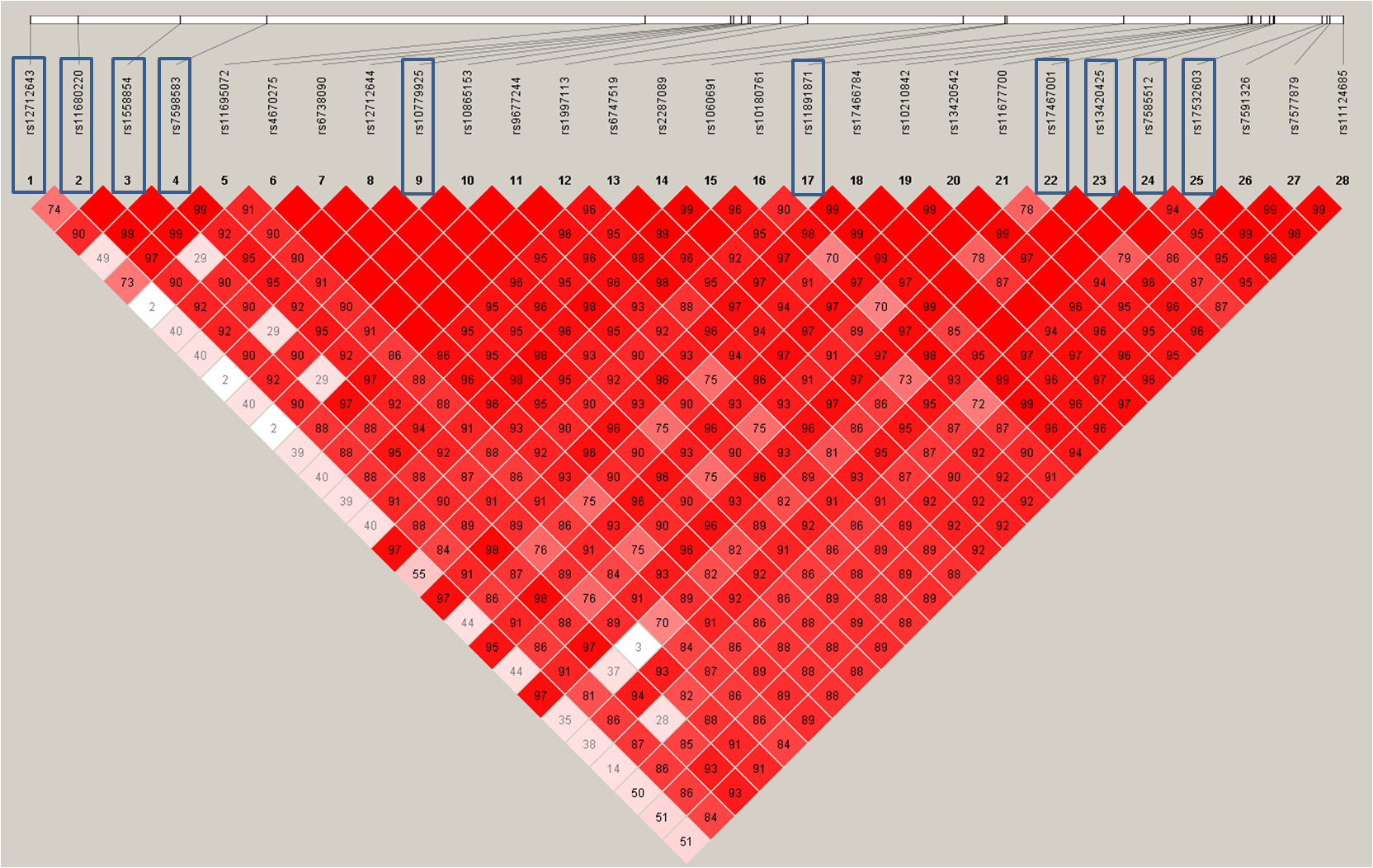


**References**

1. Barrett JC, Fry B, Maller J, Daly MJ (2005) Haploview: analysis and visualization of LD and haplotype maps. Bioinformatics 21: 263-265.
