# Supplementary material for: Gene-Based Testing of Interactions in Association Studies of Quantitative Traits
Source: PLoS Genet. 2013 Feb 28;9(2):e1003321. doi: 10.1371/journal.pgen.1003321 (PMC3585009; doi:10.1371/journal.pgen.1003321)

**Figure S4. QQ-plots of marginal association testing of SNPs from the 10 genes from Table 3 for four lipid levels.**


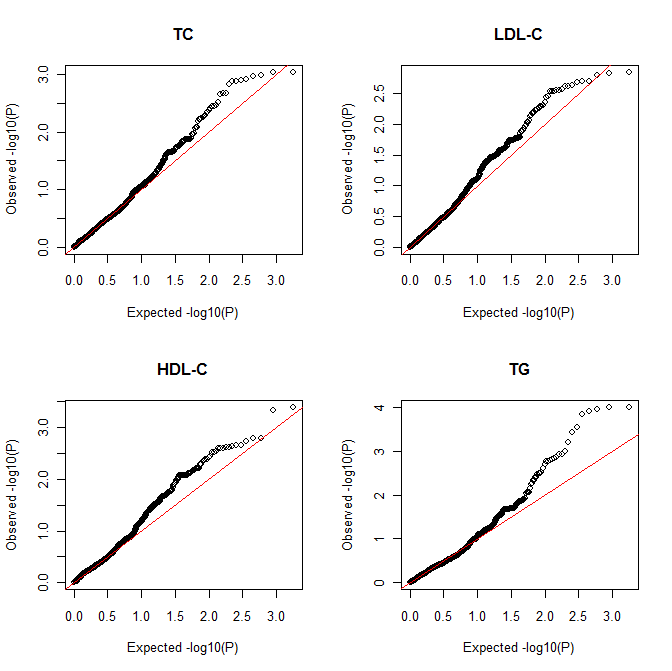

Supplement: Figure S4 — QQ-plots of marginal association testing of SNPs from the 10 genes from Table 3 for four lipid levels. (DOC) [file pgen.1003321.s004.doc]
