# Supplementary material for: Gene-Based Testing of Interactions in Association Studies of Quantitative Traits
Source: PLoS Genet. 2013 Feb 28;9(2):e1003321. doi: 10.1371/journal.pgen.1003321 (PMC3585009; doi:10.1371/journal.pgen.1003321)
Supplement: Table S1 — Empirical, simulation-based type I error rates of GGG tests using more SNPs (30 and 20) in the two genes compared to Table 1 in main text. (DOC) [file pgen.1003321.s005.doc]

**Table S1. Empirical, simulation-based type I error rates of GGG tests using more SNPs (30 and 20) in the two genes compared to Table 1 in main text.**

| *n* | α | PC | GG_minP | GG_GATES | GG_tTS | GG_tProd |
| --- | --- | --- | --- | --- | --- | --- |
| 1000 | 0.05 | 0.0504 | 0.0513 | 0.0493 | 0.0517 | 0.0491 |
|  | 0.01 | 0.0104 | 0.0113 | 0.0113 | 0.0100 | 0.0110 |
| 2000 | 0.05 | 0.0499 | 0.0503 | 0.0525 | 0.0485 | 0.0513 |
|  | 0.01 | 0.0106 | 0.0090 | 0.0107 | 0.0086 | 0.0098 |
| 3000 | 0.05 | 0.0514 | 0.0470 | 0.0506 | 0.0524 | 0.0482 |
|  | 0.01 | 0.0087 | 0.0104 | 0.0108 | 0.0116 | 0.0108 |
| 5000 | 0.05 | 0.0468 | 0.0499 | 0.0484 | 0.0496 | 0.0514 |
|  | 0.01 | 0.0099 | 0.0092 | 0.0087 | 0.0100 | 0.0098 |
