# Supplementary material for: Gene-Based Testing of Interactions in Association Studies of Quantitative Traits
Source: PLoS Genet. 2013 Feb 28;9(2):e1003321. doi: 10.1371/journal.pgen.1003321 (PMC3585009; doi:10.1371/journal.pgen.1003321)
Supplement: Table S2 — Empirical, simulation-based statistical power of GGG tests (in percentage) for low-frequency variants. (DOC) [file pgen.1003321.s006.doc]

**Table S2. Empirical, simulation-based statistical power of GGG tests (in percentage) for low-frequency variants.**

| Number of  Interacting SNPs | Type | MAFs | Effect size | *n* | Power | | | | |
| --- | --- | --- | --- | --- | --- | --- | --- | --- | --- |
|  |  |  |  |  | PC | minP | GATES | tTS | tProd |
| 1 (50-8) | U-U | .07-.09 | 0.15 | 3k | 8.2 | 6.6 | 8.2 | 8.4 | 8.6 |
|  |  |  |  | 5k | 9.0 | 9.2 | 10.3 | 10.3 | 10.1 |
| 1 (10-9) | O-O | .08-.08 | 0.15 | 3k | 9.6 | 8.6 | 9.0 | 8.2 | 8.4 |
|  |  |  |  | 5k | 9.0 | 10.4 | 11.7 | 10.0 | 10.0 |
| 3(50-8, 33-6, 25-3) | U-U | .07-.09, .08-.06, .1-.08 | 0.15 | 3k | 7.4 | 8.6 | 9.4 | 10.4 | 10.8 |
|  |  |  |  | 5k | 11.9 | 12.8 | 13.3 | 14.8 | 14.8 |
| 3 (10-9, 37-5, 13-44) | O-O | .08-.08, .1-.06, .02-.1 | 0.15 | 3k | 7.6 | 7.2 | 8.2 | 10.0 | 10.0 |
|  |  |  |  | 5k | 8.9 | 12.6 | 13.8 | 15.4 | 14.7 |
| 5 (50-8, 33-6, 25-3, 14-33, 45-15) | U-U | .07-.09, .08-.06, .1-.08, .1-.09, .1-.05 | 0.15 | 3k | 16.7 | 19.6 | 22.1 | 24.3 | 25.0 |
|  |  |  |  | 5k | 30.1 | 37.3 | 39.6 | 42.6 | 41.7 |
| 5 (10-9, 37-5, 13-44, 10-35, 37-22) | O-O | .08-.08, .1-.06, .02-.1, .08-.1, .1-.09 | 0.15 | 3k | 32.0 | 33.6 | 35.7 | 38.3 | 39.0 |
|  |  |  |  | 5k | 58.9 | 65.3 | 68.6 | 72.7 | 74.2 |
