# Supplementary material for: Gene-Based Testing of Interactions in Association Studies of Quantitative Traits
Source: PLoS Genet. 2013 Feb 28;9(2):e1003321. doi: 10.1371/journal.pgen.1003321 (PMC3585009; doi:10.1371/journal.pgen.1003321)
Supplement: Table S3 — Empirical, simulation-based type I error rates of GGG tests using external LD information. (DOC) [file pgen.1003321.s007.doc]

**Table S3. Empirical, simulation-based type I error rates of GGG tests using external LD information.**

| *n* | α | PC | GG_minP | GG_GATES | GG_tTS | GG_tProd |
| --- | --- | --- | --- | --- | --- | --- |
| 1000 | 0.05 | 0.0450 | 0.0541 | 0.0560 | 0.0624 | 0.0481 |
|  | 0.01 | 0.0098 | 0.0111 | 0.0097 | 0.0089 | 0.0100 |
| 2000 | 0.05 | 0.0473 | 0.0433 | 0.0525 | 0.0424 | 0.0566 |
|  | 0.01 | 0.0096 | 0.0114 | 0.0094 | 0.0096 | 0.0088 |
| 3000 | 0.05 | 0.0492 | 0.0512 | 0.0429 | 0.0500 | 0.0508 |
|  | 0.01 | 0.0098 | 0.0105 | 0.0110 | 0.0106 | 0.0109 |
| 5000 | 0.05 | 0.0543 | 0.0527 | 0.0480 | 0.0468 | 0.0514 |
|  | 0.01 | 0.0101 | 0.0092 | 0.0083 | 0.0108 | 0.0100 |
