# Supplementary material for: Gene-Based Testing of Interactions in Association Studies of Quantitative Traits
Source: PLoS Genet. 2013 Feb 28;9(2):e1003321. doi: 10.1371/journal.pgen.1003321 (PMC3585009; doi:10.1371/journal.pgen.1003321)
Supplement: Table S4 — Empirical, simulation-based statistical power of GGG tests (in percentage) using external LD information. Except for the test being based on external LD information (Equation 3 instead of Equation 2), the table mirrors Table 2 in the main text. (DOC) [file pgen.1003321.s008.doc]

**Table S4. Empirical, simulation-based statistical power of GGG tests (in percentage) using external LD information.** Except for the test being based on external LD information (Equation 3 instead of Equation 2), the table mirrors Table 2 in the main text.

| Interacting SNPs | Type | MAFs | Effect size | *n* | Power | | | | |
| --- | --- | --- | --- | --- | --- | --- | --- | --- | --- |
|  |  |  |  |  | PC | minP | GATES | tTS | tProd |
| 30-15 | U-U | .45-.48 | 0.15 | 1k | 13.5 | 30.3 | 32.9 | 42.6 | 44.4 |
|  |  |  |  | 2k | 24.6 | 55.7 | 57.3 | 68.7 | 68.8 |
|  |  |  |  | 3k | 46.0 | 79.3 | 80.1 | 88.7 | 86.7 |
|  |  |  |  | 5k | 68.2 | 87.9 | 91.8 | 91.5 | 92.4 |
| 30-17 | U-O | .45-.39 | 0.15 | 1k | 13.3 | 28.6 | 31.6 | 42.8 | 42.7 |
|  |  |  |  | 2k | 24.7 | 55.5 | 58.8 | 67.0 | 66.9 |
|  |  |  |  | 3k | 43.6 | 81.9 | 83.4 | 87.1 | 87.2 |
|  |  |  |  | 5k | 66.0 | 82.1 | 86.3 | 86.3 | 86.9 |
| 29-17 | O-O | .10-.39 | 0.15 | 1k | 6.5 | 9.5 | 10.2 | 7.8 | 7.9 |
|  |  |  |  | 2k | 9.7 | 15.0 | 17.3 | 12.1 | 12.7 |
|  |  |  |  | 3k | 12.6 | 25.3 | 26.9 | 18.1 | 18.7 |
|  |  |  |  | 5k | 20.5 | 48.9 | 50.3 | 32.1 | 33.3 |
| 30-15, 40-20, 48-27 | U-U | .45-.48, .41-.34, .30-.43 | 0.12 | 1k | 16.4 | 30.9 | 34.1 | 45.4 | 45.3 |
|  |  |  |  | 2k | 37.7 | 62.4 | 67.3 | 77.8 | 77.6 |
|  |  |  |  | 3k | 53.2 | 77.4 | 79.8 | 85.1 | 84.9 |
|  |  |  |  | 5k | 76.1 | 83.2 | 85.5 | 86.8 | 86.0 |
| 29-17, 39-22, 47-25 | O-O | .10-.39, .41-.38, .29-.44 | 0.12 | 1k | 17.3 | 39.5 | 43.4 | 50.1 | 50.1 |
|  |  |  |  | 2k | 33.2 | 65.6 | 68.5 | 71.6 | 71.6 |
|  |  |  |  | 3k | 49.6 | 80.9 | 81.6 | 82.8 | 82.6 |
|  |  |  |  | 5k | 81.1 | 90.8 | 91.8 | 91.8 | 91.9 |
| 10-5, 20-10, 30-15, 40-20, 48-27 | U-U | .10-.39, .10-.44, .45-.48, .41-.34, .30-.43 | 0.12 | 1k | 20.6 | 31.1 | 34.0 | 39.0 | 38.9 |
|  |  |  |  | 2k | 48.3 | 63.8 | 67.8 | 74.6 | 74.6 |
|  |  |  |  | 3k | 69.9 | 81.8 | 84.6 | 88.3 | 88.3 |
|  |  |  |  | 5k | 65.2 | 78.9 | 80.6 | 81.0 | 81.4 |
| 6-4, 19-9, 29-17,39-22, 47-25 | O-O | .12-.49, .32-.47, .10-.39, .41-.38, .29-.44 | 0.12 | 1k | 70.8 | 79.8 | 82.0 | 85.7 | 85.7 |
|  |  |  |  | 2k | 83.3 | 91.6 | 91.7 | 93.9 | 94.1 |
