# Supplementary material for: Gene-Based Testing of Interactions in Association Studies of Quantitative Traits
Source: PLoS Genet. 2013 Feb 28;9(2):e1003321. doi: 10.1371/journal.pgen.1003321 (PMC3585009; doi:10.1371/journal.pgen.1003321)
Supplement: Table S5 — Results of gene-based tests of marginal associations of the ten genes (Table 3) on four lipid levels in ARIC. (DOC) [file pgen.1003321.s009.doc]

**Table S5. Results of gene-based tests of marginal associations of the ten genes (Table 3) on four lipid levels in ARIC.**

| Trait | Gene | *P* value | | | |
| --- | --- | --- | --- | --- | --- |
|  |  | minP | GATES | tTS | tProd |
| TC | *HDAC1* | 0.792 | 0.399 | 0.235 | 0.235 |
|  | *KDM4A* | 0.818 | 0.339 | 0.420 | 0.420 |
|  | *APBB2* | 0.137 | 0.137 | 0.127 | 0.135 |
|  | *HIST1H3F* | 0.477 | 0.475 | 0.496 | 0.581 |
|  | *NEDD9* | 0.256 | 0.256 | 0.158 | 0.158 |
|  | *HDAC2* | 0.920 | 0.300 | 0.381 | 0.381 |
|  | *RPS6KA2* | 0.191 | 0.092 | 0.132 | 0.200 |
|  | *SMAD3* | 0.811 | 0.306 | 0.543 | 0.543 |
|  | *APP* | 0.689 | 0.401 | 0.856 | 0.637 |
|  | *MAPK1* | 0.172 | 0.172 | 0.126 | 0.129 |
| LDL-C | *HDAC1* | 0.298 | 0.298 | 0.234 | 0.234 |
|  | *KDM4A* | 0.861 | 0.335 | 0.405 | 0.405 |
|  | *APBB2* | 0.159 | 0.142 | 0.052 | 0.054 |
|  | *HIST1H3F* | 0.512 | 0.273 | 0.573 | 0.344 |
|  | *NEDD9* | 0.380 | 0.233 | 0.153 | 0.153 |
|  | *HDAC2* | 0.866 | 0.339 | 0.377 | 0.377 |
|  | *RPS6KA2* | 0.345 | 0.119 | 0.061 | 0.067 |
|  | *SMAD3* | 0.116 | 0.116 | 0.240 | 0.268 |
|  | *APP* | 0.959 | 0.176 | 0.912 | 0.912 |
|  | *MAPK1* | 0.312 | 0.238 | 0.244 | 0.187 |
| HDL-C | *HDAC1* | 0.803 | 0.387 | 0.257 | 0.257 |
|  | *KDM4A* | 0.927 | 0.268 | 0.421 | 0.421 |
|  | *APBB2* | 0.055 | 0.054 | 0.025 | 0.028 |
|  | *HIST1H3F* | 0.505 | 0.480 | 0.499 | 0.564 |
|  | *NEDD9* | 0.358 | 0.358 | 0.142 | 0.142 |
|  | *HDAC2* | 0.721 | 0.452 | 0.356 | 0.356 |
|  | *RPS6KA2* | 0.295 | 0.088 | 0.104 | 0.126 |
|  | *SMAD3* | 0.651 | 0.315 | 0.541 | 0.541 |
|  | *APP* | 0.605 | 0.308 | 0.527 | 0.480 |
|  | *MAPK1* | 0.211 | 0.211 | 0.185 | 0.182 |
| TG | *HDAC1* | 0.728 | 0.478 | 0.231 | 0.231 |
|  | *KDM4A* | 0.980 | 0.198 | 0.423 | 0.423 |
|  | *APBB2* | 0.168 | 0.168 | 0.161 | 0.172 |
|  | *HIST1H3F* | 0.469 | 0.469 | 0.461 | 0.557 |
|  | *NEDD9* | 0.461 | 0.333 | 0.142 | 0.142 |
|  | *HDAC2* | 0.958 | 0.259 | 0.377 | 0.377 |
|  | *RPS6KA2* | 0.026 | 0.023 | 0.098 | 0.115 |
|  | *SMAD3* | 0.475 | 0.475 | 0.468 | 0.480 |
|  | *APP* | 0.610 | 0.427 | 0.423 | 0.847 |
|  | *MAPK1* | 0.211 | 0.178 | 0.157 | 0.153 |
