# Supplementary material for: Gene-Based Testing of Interactions in Association Studies of Quantitative Traits
Source: PLoS Genet. 2013 Feb 28;9(2):e1003321. doi: 10.1371/journal.pgen.1003321 (PMC3585009; doi:10.1371/journal.pgen.1003321)
Supplement: Text S1 — Derivations of Equations (2) and (3). (DOC) [file pgen.1003321.s010.doc]

**Supporting Text S1**

**Derivation of Equation (2)**

Under the null hypothesis and with a relatively large sample size, *T*_11_ and *T*_12_ follow approximately a standard normal distribution. Thus,

**Derivation of Equation (3)**

As$S_{1}^{1}$ and $S_{2}^{1}$ in gene 1 are independent from $S_{1}^{2}$ and $S_{2}^{2}$ in gene 2,

$$\begin{matrix} Cov\left(S_{1}^{1}S_{1}^{2}, S_{2}^{1}S_{2}^{2}\right) \\ =E\left(S_{1}^{1}S_{1}^{2}S_{2}^{1}S_{2}^{2}\right)-E\left(S_{1}^{1}S_{1}^{2}\right)E\left(S_{2}^{1}S_{2}^{2}\right) \\ =E\left(S_{1}^{1}S_{2}^{1}\right)E\left(S_{1}^{2}S_{2}^{2}\right)-E\left(S_{1}^{1}\right)E\left(S_{1}^{2}\right)E\left(S_{2}^{1}\right)E\left(S_{2}^{2}\right) \\ =\left(\mu_{11}\mu_{12}+r_{1}s_{11}s_{12}\right)\left(\mu_{21}\mu_{22}+r_{2}s_{21}s_{22}\right)-\mu_{11}\mu_{12}\mu_{21}\mu_{22} \\ =r_{1}r_{2}s_{11}s_{12}s_{21}s_{22}+r_{1}s_{11}s_{12}\mu_{21}\mu_{22}+r_{2}\mu_{11}\mu_{12}s_{21}s_{22} \end{matrix}$$

$$\begin{matrix} Var\left(S_{1}^{1}S_{1}^{2}\right) \\ =E\left(S_{1}^{1}S_{1}^{2}S_{1}^{1}S_{1}^{2}\right)-E\left(S_{1}^{1}S_{1}^{2}\right)E\left(S_{1}^{1}S_{1}^{2}\right) \\ =E\left(S_{1}^{1}S_{1}^{1}\right)E\left(S_{1}^{2}S_{1}^{2}\right)-E\left(S_{1}^{1}\right)E\left(S_{1}^{2}\right)E\left(S_{1}^{1}\right)E\left(S_{1}^{2}\right) \\ =\left(\mu_{11}^{2}+s_{11}^{2}\right)\left(\mu_{21}^{2}+s_{21}^{2}\right)-\mu_{11}^{2}\mu_{21}^{2} \\ =s_{11}^{2}s_{21}^{2}+\mu_{11}^{2}s_{21}^{2}+s_{11}^{2}\mu_{21}^{2} \end{matrix}$$

Similarly,

$$Var\left(S_{2}^{1}S_{2}^{2}\right)=s_{12}^{2}s_{22}^{2}+\mu_{12}^{2}s_{22}^{2}+s_{12}^{2}\mu_{22}^{2}$$

Then,

$$\begin{matrix} Cor\left(S_{1}^{1}S_{1}^{2}, S_{2}^{1}S_{2}^{2}\right) \\ =\frac{Cov\left(S_{1}^{1}S_{1}^{2}, S_{2}^{1}S_{2}^{2}\right)}{\sqrt{Var(S_{1}^{1}S_{1}^{2})Var(S_{2}^{1}S_{2}^{2})}} \\ =\frac{r_{1}r_{2}s_{11}s_{12}s_{21}s_{22}+r_{1}s_{11}s_{12}\mu_{21}\mu_{22}+r_{2}\mu_{11}\mu_{12}s_{21}s_{22}}{\sqrt{\text{(}s_{11}^{2}s_{21}^{2}+\mu_{11}^{2}s_{21}^{2}+s_{11}^{2}\mu_{21}^{2}\text{)(}s_{12}^{2}s_{22}^{2}+\mu_{12}^{2}s_{22}^{2}+s_{12}^{2}\mu_{22}^{2}\text{)}}} \end{matrix}$$
